# Target repositioning using multi-layer networks and machine learning: The case of prostate cancer

**DOI:** 10.1016/j.csbj.2024.06.012

**Published:** 2024-06-15

**Authors:** Milan Picard, Marie-Pier Scott-Boyer, Antoine Bodein, Mickaël Leclercq, Julien Prunier, Olivier Périn, Arnaud Droit

**Affiliations:** a Molecular Medicine Department, CHU de Québec Research Center, Université Laval, Québec, QC, Canada; b Digital Transformation and Innovation Department, L′Oréal Advanced Research, Aulnay-sous-bois, France

**Keywords:** Multi-omics, Target prioritization, Drug discovery, Disease signature, Random walk, Machine learning

## Abstract

The discovery of novel therapeutic targets, defined as proteins which drugs can interact with to induce therapeutic benefits, typically represent the first and most important step of drug discovery. One solution for target discovery is target repositioning, a strategy which relies on the repurposing of known targets for new diseases, leading to new treatments, less side effects and potential drug synergies. Biological networks have emerged as powerful tools for integrating heterogeneous data and facilitating the prediction of biological or therapeutic properties. Consequently, they are widely employed to predict new therapeutic targets by characterizing potential candidates, often based on their interactions within a Protein-Protein Interaction (PPI) network, and their proximity to genes associated with the disease. However, over-reliance on PPI networks and the assumption that potential targets are necessarily near known genes can introduce biases that may limit the effectiveness of these methods. This study addresses these limitations in two ways. First, by exploiting a multi-layer network which incorporates additional information such as gene regulation, metabolite interactions, metabolic pathways, and several disease signatures such as Differentially Expressed Genes, mutated genes, Copy Number Alteration, and structural variants. Second, by extracting relevant features from the network using several approaches including proximity to disease-associated genes, but also unbiased approaches such as propagation-based methods, topological metrics, and module detection algorithms. Using prostate cancer as a case study, the best features were identified and utilized to train machine learning algorithms to predict 5 novel promising therapeutic targets for prostate cancer: IGF2R, C5AR, RAB7, SETD2 and NPBWR1.

## Graphical Abstract

**Figure:**
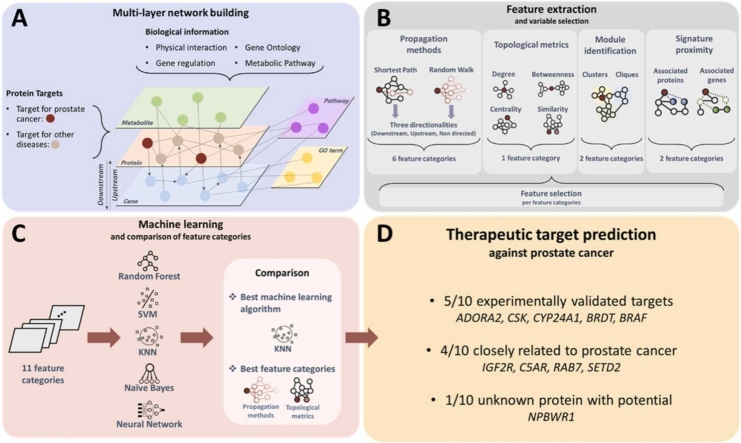
The workflow of the target repositioning strategy included three steps. Step 1 (A): a multi-layer network was built to integrate all relevant biological information related to prostate cancer and the known therapeutic targets for prostate cancer were retrieved. Step 2 (B): using four different graph-based approaches, multiple features were extracted from the network. The most predictive features for each feature category were then identified through variable selection. Step 3 (C): machine learning algorithms were trained using the selected features to predict therapeutic targets and evaluation of the best algorithms and feature categories was conducted. Finally (D): ten therapeutic targets for prostate cancer were predicted and validated using literature and network-based interpretation.

## 1 Introduction

Traditional drug development is a costly and time-consuming process which requires expertise in the fields of biology, chemistry, and pharmacology. To address these limits, drug repositioning has been proposed in recent years, which relies on finding new indications for known drugs [1]. This strategy offers a significant advantage since all drugs currently on the market have successfully completed the required clinical test phases, thereby guaranteeing their low toxicity profiles [2]. Furthermore, large amounts of information have already been gathered for these drugs which can be exploited to predict novel indication such as their binding targets, side effects, drug interactions, and pharmacological properties [3].

Similarly, there has been a growing interest in target repositioning, the strategy of identifying and validating known therapeutic targets for other diseases [4]. Therapeutic targets are proteins that can be targeted by drug-like molecules to induce a therapeutic effect. Target repositioning has several advantages over traditional drug repositioning, mainly because the initial selection of therapeutic targets is generally the first and most important part of drug discovery [5]; drug candidate failures and off-target interactions are often due to the wrong selection, or the misidentification of true therapeutic targets [6], [7]. Therefore, the correct identification of therapeutic targets for a disease should be prioritized and target repositioning fills that purpose while having the same advantages of drug repositioning, mainly the existence of known interacting drugs with low toxicity profiles. Moreover, the identification of novel therapeutic targets involved in different molecular pathways can lead to the development of new treatment options and potential drug synergies [8]. This strategy is even more valuable for complex diseases like cancer, where drug resistances often emerge and new means of tackling the tumor must be found [9].

Biological networks have emerged as powerful tools for studying complex biological models as they can seamlessly integrate heterogeneous information about genes or proteins while providing interpretable solutions [10]. These benefits make biological networks particularly well-suited for target repositioning which might necessitates various level of omics to accurately predict therapeutic potential. Their structures are usually based on interactome information to represent physical or functional interactions between molecules within cells. To date, most target repositioning research have used Protein-Protein Interactions (PPI) networks [11] to study biological functions and infer new potential targets [12], [13], [14], [15], often by harnessing structural information within the network, such as hubness, betweenness, or centrality measures. These methods rely solely on the network’s topology and can be summarised as detection of essential nodes, which often translates to influential proteins that could be targeted for treatment.

Recently, the creation of more powerful network approaches has further improved target prediction, especially with the integration of disease specific omics data to enrich the network with relevant information. Altered pathways and perturbated gene expression are often the causes or symptoms of disease manifestation and progression, therefore targeting these dysregulated mechanisms will often correct the cellular biology and eventually treat the disease. It has been demonstrated that gene expression alone is insufficient for accurately predicting therapeutic targets as there is poor overlap between Differentially Expressed Genes (DEG) linked to a disease and known therapeutic targets. Nevertheless, the integration of gene expression within a network can still lead to more accurate predictions [16]. It is generally assumed that new potential protein targets are near genes associated with the disease. These genes are often dysregulated in the disease, driving disease progression and treatment resistances. Therefore, potential targets should also be near disease-associated nodes in the network. This proximity can then be calculated in different ways, with many feature engineering methods being proposed such as random walks [17], shortest paths [18], or new centrality measures [16], but the best method to prioritize targets based on their proximity to dysregulated nodes has yet to be identified. A comparison of approaches made by Emig *and al*. (2013) [19] revealed that local and global topologies were equally important for accurate target prediction. However, strong variabilities existed between diseases and the authors eventually opted for a consensus approach to calculate proximity to dysregulated genes including propagations, neighborhood scoring and connectivity.

Even more recently, embedded features calculated through deep learning models have been used with success in target repositioning efforts [20], [21]. Compared to traditional feature engineering, the automation of the process and its ability to capture complex relationships in the network are key advantages and embedded features might give better accuracy that engineered ones. However, embedded features lack interpretability, which makes it difficult to understand why and how a potential target was predicted, thus triggering lack of trust into new unvalidated results.

Overall, the published methods about target repositioning are limited by several factors. They mainly depend on PPI networks and their structure, which were shown to be prone to technical errors and research biases making their topology less reliable [22], [23], [24], [25]. Additional information could be integrated to PPI networks for better understanding of protein function and reliability [26], [27]. Moreover, diseases are mostly represented by their gene expression alone, while more knowledge is usually accessible about the disease such as mutated genes or methylation patterns, which might also help in better characterising known targets, and eventually predict new ones. Finally, most of the target prediction approaches assume that potential targets are near predefined nodes, either DEGs or existing therapeutic targets. This assumption generally holds true but might fall short in some cases including diseases with few known targets or DEGs. It is also possible that proximity to DEGs is not the most informative and that the proximity to other signatures such as mutated genes or structural variants is more predictive. Preferably, the approach should not be biased towards proximities with specific nodes and should consider each node in the network.

In this study, we proposed an unbiased approach which only considers the known therapeutic targets of a given disease, and empirically evaluate the best methods for identifying novel protein candidates using machine learning algorithms. Building upon prior research efforts [12], [19], [28], this study distinguishes itself in two key aspects. First, additional information including gene regulations, metabolite interactions, and annotated knowledge was incorporated into PPI networks to better model cellular processes, alongside various gene signatures associated with the disease such as DEGs, mutated genes, structural variants, and Copy Number Alterations (CNA). Second, meaningful features were extracted using various network-based analytical methods, including proximity to disease-associated genes, as well as unbiased approaches based on propagation, topological metrics, and module detection. While these methods have been extensively applied in drug repositioning scenarios [29], [30], [31], [32], [33], [34], [35], [36], [37], [38], their effectiveness in target repositioning remains to be better characterised. Using the extracted features, various machine learning algorithms were then trained, and the predictive power of each approach was evaluated. Additionally, the usefulness of each layer in the multi-layer network and the importance of network directionality were also investigated.

We applied our approach to a case study: the repositioning of new targets for prostate cancer, one of the leading causes of cancer-related death among men in the world [39]. Currently, the leading treatment against prostate cancer is Androgen Deprivation Therapy (ADT), which targets a handful of proteins involved in the androgen axis such as the Androgen Receptor, CYP17A1 or GnRH receptors [40]. However, many patients eventually relapse on ADT due to the development of resistance and evasion mechanisms [41], [42], [43], [44]. Targeting new pathways in prostate cancer could lead to the discovery of novel treatments, and existing therapeutic targets for other disease could be repurposed to achieve that goal. Our approach eventually predicted five new therapeutic targets that could potentially lead to new treatment options for patients with prostate cancer. A R package to reproduce the approach has been developed and made available at https://github.com/MilanPicard/DiscoNet. It supports graph-based extraction of features and variable selection methods.

## 2 Material and methods

### 2.1 Workflow of the target repositioning strategy

To repurpose therapeutic targets for prostate cancer, our strategy relied on three main steps (Graphical Abstract). The first step involved gathering a large quantity of biological data and integrating it into a multi-layer network modeling prostate cancer. This data encompassed physical interactions between proteins and metabolites, genetic regulations involving transcription factors, micro-RNA, and genes, as well as functional annotations of these molecules using Gene Ontology and Metabolic Pathways. This information is described in more detail in Section 2.3. Additionally, known prostate cancer targets were identified from the literature, defined as proteins that can be targeted by known therapeutic drugs to treat the disease and its symptoms, hence labelling them as positive targets. Other protein targets within the network were labelled as negatives, representing potential candidates for repositioning (Section 2.2.). The second step entailed employing four different network-based approaches to analyze the multi-layer network and extract relevant features for each target (Section 2.5.). These approaches included propagation-based methods, topological metrics, module detection, and proximity to nodes signatures, defined as genes or proteins associated with prostate cancer (details in Section 2.4.). Then, variable selection was done to retain only the most predictive features which can give valuable insights into the importance of the different disease signatures of the various network layers (Section 3.2. and 3.3.). Finally, the third step involved leveraging the selected features to train machine learning algorithms to predict new therapeutic targets for prostate cancer (Section 3.4.). This allowed us to directly compare the predictiveness of the different feature categories extracted from the network (Section 3.5.) and, based on the most discriminative ones, predict new therapeutic targets for prostate cancer (Section 3.6.).

### 2.2 Known therapeutic targets for prostate cancer

Therapeutic targets were defined as proteins that can be targeted by drugs to induce therapeutic benefits against a disease. Existing therapeutic targets for prostate cancer were identified so that machine learning algorithms can train to recognise these targets as positive and eventually predict new ones. To retrieve therapeutic targets for prostate cancer, drugs currently administered or investigated for prostate cancer were searched and manually selected from clinicaltrials.gov using keywords “prostate cancer” and “CRPC, advanced prostate cancer”, and in DrugBank [45] using keywords such as “prostat” AND (“cancer” OR “neoplasm” OR “tumor” OR “carcinoma”). A total of 120 prostate cancer drugs were found. Then, using the curated drug-target interactions from the Therapeutic Target Database [46] and DrugBank, the targets of these drugs were retrieved. A total of 110 proteins targets were identified, for the remaining of the paper these proteins were defined as the known therapeutic targets for prostate cancer, considered as the positive class. The negative class was constituted of all other protein targets.

### 2.3 Multi-layer network construction

To integrate the biological information necessary for this research, a multi-layer network was built using five different layers: gene, protein, metabolite, molecular pathway, and Gene Ontology. The interactions between and within layers can be non-directed or directed (downstream or upstream) depending on the type of interaction. Precise details for each interaction type are given in the following paragraphs. Only interactions with the highest confidence as annotated by the providing database were retrieved to ensure that the multi-layer network is reliable. Proteins IDs were all converted were converted to Uniprot IDs and genes IDs to Gene Symbol using the Bioconductor version 3.14 package *org.Hs.eg.db* [47]. Metabolites were converted to PubChem ID using the PubChem Identifier Exchange Service (https://pubchem.ncbi.nlm.nih.gov/idexchange/idexchange.cgi). Due to the ID standardization, the layers within the network can interact together. The network is available as an edge list in Supplementary data.

#### 2.3.1 Protein-protein interactions

The Protein-protein interactions (PPI) network was built by integrating interactions from HURI(v1) [48], the interactions from the STRING (v11.5) [49] database with an “experiments” score higher or equal than 700, all interactions with strong experimental evidence from APID(v1) [50], and all interactions with “High” confidence from HitPredict (v4, 30/08/2021) [51]. These interactions produce non-directed edges between protein nodes.

#### 2.3.2 miRNA-Gene interactions

As micro-RNAs can regulate many different genes, miRNA-gene relationships were retrieved only when strong confidence existed to support their interaction. From miRTarBase (v9.0) [52] miRNA-gene interactions with “strong” experimental evidence were retrieved, as well as interactions with at least one experimental validation that are also predicted (binding probability > 95 %) in the miRWalk database (v2 Release 2022) [53] to ensure that they are of high confidence. These interactions produce directed edges (miRNA → coding gene) between nodes within the gene layer.

#### 2.3.3 TF-Gene interactions

All experimentally validated interactions between transcription factors and their target genes were retrieved from RegNet (v2019) [54], HTRI (v2012) [55], TRRUST (v2018) [56], as well as interactions with an “A” level of confidence from Dorothea (v1.6.0) [57] and interactions with a “level 1” confidence from TransmiR [58]. These interactions produce directed edges (TF → coding gene or miRNA) between protein nodes to gene nodes, resulting in downstream information flux.

#### 2.3.4 Coding gene-Protein interactions

To represent the translation of genes to protein products, interactions from coding genes to their respective proteins were added when possible, using the Bioconductor package *org.Hs.eg.db.* These interactions produce directed edges (coding gene → protein) between gene nodes to protein nodes, resulting in upstream information flux.

#### 2.3.5 Protein-Metabolites interactions

Interactions between metabolites and proteins were retrieved in October 2021 from KEGG [59], Reactome [60] and WikiPathways [61] using the R package *graphite* v1.40 [62]. These interactions produce non-directed edges between protein and metabolite nodes.

#### 2.3.6 Gene Ontology and Molecular Pathways

Relationships between Genes and Gene Ontologies were retrieved using the R package *GofuncR* (v1.14) [63]. Involvement of proteins, genes and metabolites in molecular pathways were determined using the R package *graphite* based on KEGG, Reactome and WikiPathways, retrieved in October 2021. This information was connected through non-directed edges to other nodes.

#### 2.3.7 Node filtering

Current therapies against prostate cancer often work by targeting proteins and pathways within specific tissues, therefore only genes expressed in these tissues were kept in the network as to not over complexify its structure. Proteins derived from these genes were kept as well. Two databases were exploited to retrieve tissue-specific gene expression. In the Human Proteome Atlas (v21.0) [64], genes with transcripts expressed in at least one of the following tissues were kept: prostate, prostate cancer, adrenal gland, and testes. Additionally, prostate cancer transcriptomics were retrieved from Archs4 (v11) [65] and transcripts with Transcripts Per Million (TPM) strictly higher than 1 in at least a hundred samples were kept as well. To limit technical errors and missing information, the gene selection was based on tissue expression profiles from both databases (N = 17 574).

### 2.4 Retrieving prostate cancer specific signatures

In addition to the multi-layer network, biological data specific to prostate cancer was retrieved to build disease signatures, which will eventually be used to construct signature-based features (Section 2.4.4). These signatures represent association between genes and different facets of prostate cancer like mutations or epigenetic changes, and proximities to these genes can be interesting features for target repositioning. These signatures were retrieved from online databases and not produced by us through additional data analysis. From a repository of cancer genomic datasets (CbioPortal, retrieved in April of 2023 [66]), gene signatures were retrieved including mutated genes in castration-resistant prostate cancer (frequency >= 5 %, N = 56), Structural Variants (SV) (frequency ≥ 1 %, N = 24) which are alterations in DNA sequence such as inversions or translocations, and Copy Number Alterations (CNAs) (frequency ≥ 12 %, N = 108) which are changes in the number of copy of a genomic elements. In addition, we retrieved genes differentially expressed in prostate cancer compared to healthy individuals, including over-expressed genes (N = 266) and under-expressed genes (N = 1466) from a comprehensive database of prostate cancer transcriptomics datasets (PcaDB [67]) using a log Fold Change greater than 2 and an adjusted p-value lower than 0.05 as the selection criteria. Finally, these five gene sets were aggregated into three additional ones to latter assess their interplay: one containing both over- and under-expressed genes (*Combined DEG*, N = 1732), another comprising all genetic modifications (*Combined alteration*: mutations+ SV+ CNA, N = 184), and a third encompassing all sets (*Combined all*, n = 1904). This resulted in a total of eight distinct sets of genes associated with prostate cancer. The overlap between these signatures and the different network`s layer is provided in Supplementary Materials Section S5.

### 2.5 Feature extraction

A total of 11 types of features were extracted from the multi-layer network for each target, representing 4 different categories including propagation-based features, topological measures, module-based features, and signature-based features.

#### 2.5.1 Propagation-based features

Propagation-based methods were used to calculate the distance between each potential target and any other single nodes in the multi-layer network. To test if the directionality of edges impacted the results, three variations of propagation were considered: *Downstream* (A → B) which represents the influence of a target upon another node, *Upstream* (A ← B) which represents the influence of a node on a target, and lastly *Non directed* (A—B) which explores the network unhindered by edge directionality and represents general proximity between a target and a node. For each type of propagation, two methods were used to calculate distances, *shortest paths* using the *distances* function of the *igraph* R package [68] and *Random Walk with Restart* using a code inspired by the R package *RandomWalkRestartMH* [69] modified as to allow random walks on directed networks. Both methods were tested to evaluate predictive and behavioral differences between them. In total, 6 categories of features were created by propagation (two methods/three directionalities).

#### 2.5.2 Topological metrics

Topological measures such as centrality or betweenness are often used to assess the importance of a node in a biological network, and ultimately in a disease. Additionally, simple similarity measures between two proteins based on their common neighboring nodes can reveal similar biological functions, and involvement in related molecular pathways which is pertinent to predict their therapeutic potential. Topological measures were calculated for each target using the *igraph* R package and include 1) Degree: number of edges going out and in of a node, 2) Closeness: inverse of the sum of distances to all other vertices, 3) Harmonic centrality: inverse of the mean distance to all other vertices, 4) Eigenvector centrality: the first eigenvector of a node based on the adjacency matrix of the network. It can be interpreted as the influence of a node on the network, 5) Betweenness: roughly the number of times a node acts as a bridge along shortest paths between two nodes, thus showing how much a node is a bottleneck within a network, 6) Eccentricity: shortest distance from the farthest other node, and 7) Similarity: measure the similarity between each target and all other nodes in a graph using the *similarity.invlogweighted* function of the *igraph* R package, which considers the number of common neighbors between two nodes, weighted by their degrees. All these features were grouped into one additional category of features extracted from the network.

#### 2.5.3 Module-based features

Module-based methods represent proximities between targets and specific groups of nodes identified in the network using topological analysis. Modules often represent functional units within biological pathways and proteins involved in these node communities can be interesting therapeutic targets. Two types of modules were identified, clusters which are nodes closely connected together and cliques which are nodes completely connected. Most cluster identification algorithms are not designed for multi-layer networks including the ones employed in this study [70] and were therefore applied only to the protein-protein interaction layer. Clustering algorithms available through the *igraph* R package were utilized: *cluster_leading_eigen*, *cluster_louvain* (with different resolutions of 1, 1.5 and 2, by changing the resolution parameter) and *cluster_walktrap* (with different steps of 4 and 5 by changing the steps parameter), with other parameters left to default settings. Additionally, we applied K1 and M1 methods from the GitHub repository MONET which implement kernel clustering and multiresolution clustering respectively, with default parameters [71]. Due to the high number of cliques in the multi-layer network, only a smaller number of cliques were retained using the *max_cliques* function of the *igraph* R package, and the following criteria: i) a node size between 15 and 50, ii) the clique is maximal meaning it cannot be extended by adding another node, and to insure biological interpretability iii) each protein in a clique must be linked to at least one molecular pathway as defined by the annotated knowledge present in the network. These filters ensure cliques of similar sizes that can later be biologically interpretated. Using several module identification algorithms ensured that the module-based features did not overly rely on the efficacity of only one algorithm, as their performances can vary between biological networks. The distance between targets and each module was then calculated based on the distance measure proposed by Guney *and al*. (2016) [72], which is the average of the total shortest path distances between the node and each node in the module. This yielded two additional feature categories (cluster and clique).

#### 2.5.4 Signature-based features

Signature-based methods assessed proximities between protein targets and genes associated with prostate cancer according to the literature. These genes include mutated genes, Differentially Expressed Genes (DEGs), as well as genes impacted by Copy Number Alterations (CNAs), and structural variants (See Section 2.3). These gene signatures were mapped as attributes on the gene layer. Additionally, after conversion of these signatures to Uniprot IDs, their corresponding protein signatures were also mapped to the protein layer. Distances between a target and a set of nodes associated with prostate cancer were also calculated using the average of the total shortest path distances between a target and each node in the signature set. Furthermore, because signatures can be quite large (n > 100) and thus less biologically interpretable, smaller sets of nodes associated with prostate cancer were also delineated. These subsets were identified by intersecting each signature set with each module previously identified (Section 2.4.3), thereby forming several smaller groups of nodes within the same module that were all associated with prostate cancer. Subsequently, distances between targets and these refined node sets were computed using the previously described distance measure. This approach yielded two other feature categories (Proximity to gene signatures, and Proximity to protein signatures).

### 2.6 Feature Selection and data splitting

Once all features were extracted, protein targets were kept only if they were verified targets using the ‘Tclin’ or ‘Tchem’ denomination of the Pharos database [73]. These denominations indicates that a protein can bind to either approved drugs or small molecules with activities listed in ChEMBL [74]. The other proteins were left out of further analysis. The dataset was then split into a training set containing 80 % of targets and a test set containing the remaining 20 % using stratified random sampling. Afterwards, a multi-step feature selection was employed to reduce the number of features and select the most predictive ones. This selection was done separately for each of the 11 feature categories previously extracted to allow independent evaluation of their performances. The first step was a broad variable selection based on high Information Gain and low correlation with other features. Then, using the remaining features, Adaptative LASSO and a Random Forest importance measure were applied. Using a hundred different bootstraps of training data to ensure stability and robustness, the union of features selected by both methods across the different bootstraps formed the final set of features. More information is given in the next sections.

#### 2.6.1 Information Gain and correlation for feature selection

Information gain refers to the reduction in entropy achieved by splitting a dataset according to the values of a particular variable [75]. For each feature, the information gain was calculated 15 times through bootstrapping with replacement using the *InfoGainAttributeEval* function from the R package *Rweka* [76]. The average over all iterations denotes the final information gain of a feature. Features with information gain higher than a specific threshold were kept. This threshold represents the upper bound on Tukey’s fences method for outlier detection on the null distribution of Information Gain scores, generated by applying the graph-based methods (Section 2.4) on a random network. The distributions obtained and thresholds used are available in Supplementary data Section S1. Then, using the *findCorrelation* function of the R package *caret* [77] over correlated features were removed using a correlation cutoff of 95 %.

#### 2.6.2 Adaptative LASSO and random forest for feature selection

To ensure robustness in the features selection process, adaptive LASSO (Least-Absolute Shrinkage and Selection Operator) was repeated 80 times with 10-fold stratified cross-validation. Features selected by adaptive LASSO in at least 80 % of repetitions were kept using the r package *glmnet* [78]. Simultaneously, another feature selection method was performed using out-of-bag (OOG) accuracy during a random forest training process implemented using the r package *randomForest* [79]. Variable importance was calculated as the average importance of each feature over 100 repetition of 10-fold stratified cross-validation and the best features were kept based on the “elbow method”. For each 11 feature categories, the consensus of both variable selection methods was returned as the final selection of features.

### 2.7 Machine learning methods

Several machine learning algorithms were exploited to predict new therapeutic targets, including Random forests, weighted Support-Vector Machines, Naïve Bayes, weighted k-nearest neighbors, and Artificial Neural Networks, implemented using the r packages *randomForest* [79]*, e1071* [80]*, naivebayes, kknn,* and *h2o* [81]. Hyperparameter tuning was done using 10 repetitions of stratified 10-fold cross validation on the training set. Performances during training were evaluated with Matthiew Correlation Coefficient (MCC) to consider class imbalance. The hyperparameters tuned for each model are available in Supplementary data Section S3.

### 2.8 Final prediction of novel therapeutic targets

To predict novel therapeutic targets (Section 3.6), the entire pool of targets must be considered, thus training and test set were merged. On the resulting dataset, through a 100 repetition of 10-fold cross validations, the targets predicted positive at least 50 times were classified as positives by the model. This process was applied to each feature type. Then, the final prediction was based on the consensus predictions of the most predictive feature types (Section 3.5) to ensure reliable predictions.

## 3 Results

### 3.1 Feature extraction from a multi-layer network and variable selection

Before building machine learning classifiers to predict novel therapeutic targets for prostate cancer, a multi-layer network was built, and various features were extracted from its topology to characterize each target. The network was constructed using online repositories and databases (Section 2.3), it integrated physical interactions between genes, proteins, and metabolites, as well as functional annotations between molecules and biological knowledge represented as nodes in the network. The network was made of five different layers: gene (+miRNA), protein, metabolite, Gene Ontology, and Molecular Pathway. Subsequently, four network-based approaches were utilized to extract a total of 460 832 distinct features. These approaches included propagation-based methods (Random Walk with Restart and Shortest Path), Topological Metrics (common centrality measures, and network-based similarity between nodes), Module Detection, and Signature-Based methods (proximity to nodes associated with prostate cancer). To assess the efficacy of each approach in producing predictive features, as well as to constrain the number of features later provided to machine learning algorithms, a robust variable selection process was implemented (Section 2.6). Ultimately, 2 980 features across all feature categories were identified as the most discriminative based on their ability to separate positive and negative targets (defined in Section 2.2) (Table 1).

**Table 1:** Number of features extracted and selected from the multi-layer network by categories.

Feature type	Description	Features extracted	Features selected
Propagation-based	Distances between a target and other single nodes.	387 321	2 457
Module-based	Average distance between a target and a PPI module identified.	1 928	86
Signature-based	Average distance between a target and a set of nodes associated with a prostate cancer signature.	6 144	159
Topological metrics	Topological measures, and similarities between a target and other nodes.	65 439	278

### 3.2 Assessing the importance of various prostate cancer signatures

Proximity between a protein and nodes associated with a disease is one of the most common ways of identifying the protein as a suitable therapeutic target, referred as the signature-based approach. Using several types of disease signatures, 6 144 features were extracted from the network and subjected to a robust variable selection process to select only the most predictive ones. In this section, an in-depth analysis was done on the 159 predictive features selected (before machine learning prediction) to understand which types of signatures led to the highest number of predictive features. For a given signature, a higher number of selected features approximated to a greater predictive power.

**Fig. 1:**
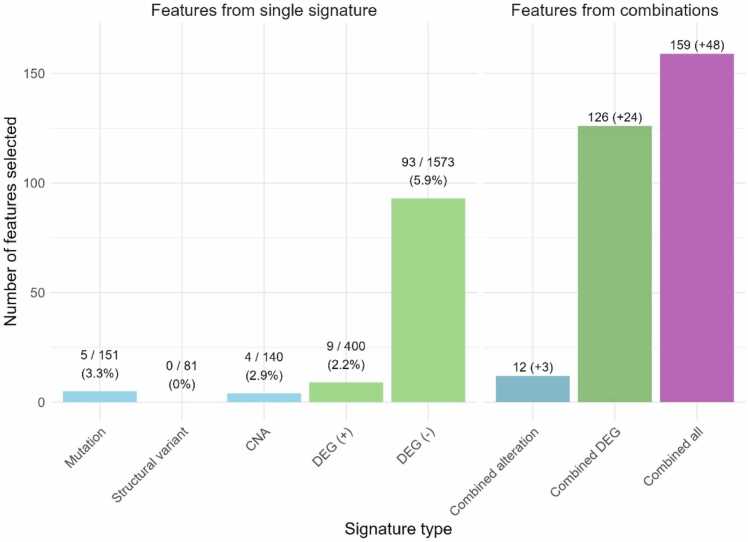
Barplot showing the number of predictive features selected by the variable selection (y-axis) for each gene signature, summed between both categories of features (Proximity to gene signatures and Proximity to protein signatures). The number of features based on single gene signatures are represented on the left side, in order: mutations, structural variants, Copy Number Alterations (CNA), over-expressed genes (DEG (+)), and under-expressed genes (DEG (-)). Additionally, the total number of features initially extracted for each signature is given as well, along with the percentage of features selected. On the right side is represented the number of features from combined sets of signatures. The first three signatures (Mutation, Structural variants, and CNA) were merged into the Combined alteration set (color: blue). DEG (+) and DEG (-) were merged into Combined DEG that represents all differentially expressed genes (color: green). And every signature was merged into Combined All, which considers all genes (color: purple). The additional predictive features created by combining signatures are noted in parenthesis.

Proximity to structural variants genes, despite having significant implications for the biology of prostate cancer and its response to treatment [82], was not found to be predictive in this study, resulting in no selected features (Fig. 1). Proximity to mutated genes, Copy Number Alterations (CNA) and Over-Expressed Genes also yielded limited numbers of predictive features (5, 4 and 9 features, respectively), which represents about 2–3 % of all features extracted from these signature types. In contrast, proximity to Under Expressed Genes emerged as more predictive, with a higher percentage of features being selected (5.9 %) and resulting in more predictive features (93). Notably, a substantial discrepancy was observed between the number of selected features associated with over-expressed genes (9) and under-expressed genes (93).

Moreover, combining signatures provided new additional predictive features compared to single signature types. *Combining Alterations* (mutation, CNA, structural variants), *Combined DEGs* (over- and under- expressed genes), and *Combined All* (union set) led to 3, 24, and 48 additional predictive features. For example, one feature made from the *Combined All* set represents the average shortest path distance from four different genes that were all associated with prostate cancer and part of the same cluster (clusters were identified with several methods presented in Section 2.5): NEK2, CDK1, ALMS1, and TPX2. These genes are fully connected in the PPI network meaning that their protein products all interact with each other. Additionally, they were shown in the literature to be involved in the regulation of the cell cycle and mitosis [83]. The distance to each of these genes separately was not determined to be useful by the feature selection process, but together they formed a small functional unit of dysregulated nodes whose proximity to potential targets was found to be useful for discriminating positive from negative targets. This group of four genes would not have been detected using a single signature as ALMS1 is mutated in prostate cancer while the other three are over-expressed. This example highlights the usefulness and biological interpretability gained by exploiting multiple prostate cancer signatures for target repositioning.

### 3.3 Assessing the importance of additional network layers

In this section, the benefits of having additional network layers were assessed. Propagation-based approaches (Random Walks with Restart (RWR) and Shortest Paths (SP)) calculated the distance between each target to every other node in the network. These distances were represented as features and the most predictive ones were selected with a robust variable selection process which assessed their ability to separate positive from negative targets (Section 2.6). Then, the usefulness of a layer was assessed using the number of predictive features (distances to its own nodes) that were selected by variable selection. For a given network layer, a higher number of selected features approximated to a greater predictive power.

**Fig. 2:**
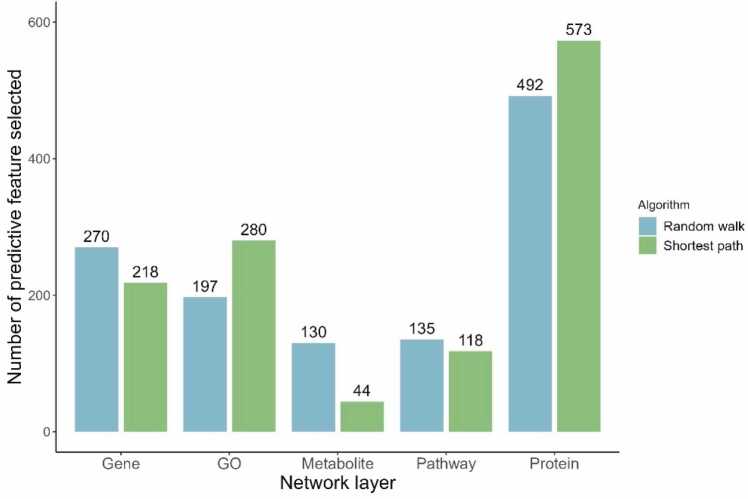
Barplot showing the number of predictive propagation-based features selected for each network layer. The higher the number of features selected, the more informative the layer is in separating positive from negative targets. Two types of algorithms were used, shortest paths and Random Walks with Restart. In this plot, the selected features are summed between the types of directionalities (*Downstream*, *Upstream*, and *Non directed*) for each algorithm. In parenthesis is shown the number of predictive features (a feature represented a distance to a single node) divided by the total number of nodes in each layer. Features based on distances to protein nodes were the most informative, but other layers also bring relevant information. The choice of algorithm did not change these results. GO: Gene Ontology layer.

For both distance methods (RWR and SP), proximities with protein nodes led to the highest number of predictive features (RWR = 492 and SP = 573, Fig. 2), revealing that PPI networks were indeed the most valuable information to characterize prostate cancer targets. The other layers comparatively returned fewer features, especially the metabolite and molecular pathway layers (which can be partly explained by their size, details in Supplementary Materials Section S4). However, the combined number of predictive features selected for the other layers (gene, GO, metabolites, and molecular pathways) were in fact higher than those from the protein layer (RWR = 732, SP = 660). Overall, these results showed that while PPI networks do contain relevant information to characterise protein targets, multi-layer networks provided more information and predictive features and therefore they should be prioritized for target repositioning. These results were similar between both propagation methods and do not change when considering the overall distance between targets and the layer considered.

### 3.4 The KNN algorithm performs best on an independent dataset

Five algorithms were trained and tuned on the training set to accurately classify positive and negative targets: Random Forest, Support Vector Machines, Naive Bayes, K-Nearest Neighbors, and an Artificial Neural Network. Their performances were averaged for all feature categories on the training and independent test dataset (Table 2). Detailed accuracy using different metrics are given in Supplementary Materials Section S6. Performances were evaluated with Matthews Correlation Coefficient (MCC) as it was shown to be better suited to evaluate binary classification performances in a class imbalance scenario [84]. A value of zero represents a random prediction and a value of one a perfect prediction.

**Table 2:** Performances of 5 machine learning algorithms using MCC (standard deviation). The best performance is represented in bold. Abb.: RF: Random Forest; SVM: Support-vector machines; NB: Naïve Bayes; KNN: k-nearest neighbors; ANN: Artificial Neural Network.

Algorithm	RF	SVM	NB	KNN	ANN
Training MCC	0.22 (0.07)	0.28 (0.15)	0.2 (0.09)	0.19 (0.07)	**0.38 (0.15)**
Test MCC	0.11 (0.05)	0.14 (0.12)	0.09 (0.06)	**0.17 (0.1)**	0.11 (0.08)
Difference	0.11	0.14	0.11	**0.02**	0.27

Most algorithms failed to generalize on new data with an average decrease of MCC of 47 % from training to test set. Nevertheless, despite the K-nearest neighbors (KNN) exhibiting poorer performances during training (MCC = 0.19), it was notably the only algorithm showing consistent generalization, with its performance maintaining stability on the test set (MCC = 0.17). KNN also exhibited the best performances on the test set compared to an average of 0.11 for all other algorithms. Because of these qualities, the KNN algorithm was the machine learning algorithm chosen for subsequent analyses.

### 3.5 Classification performances showed high variance between feature categories

**Fig. 3:**
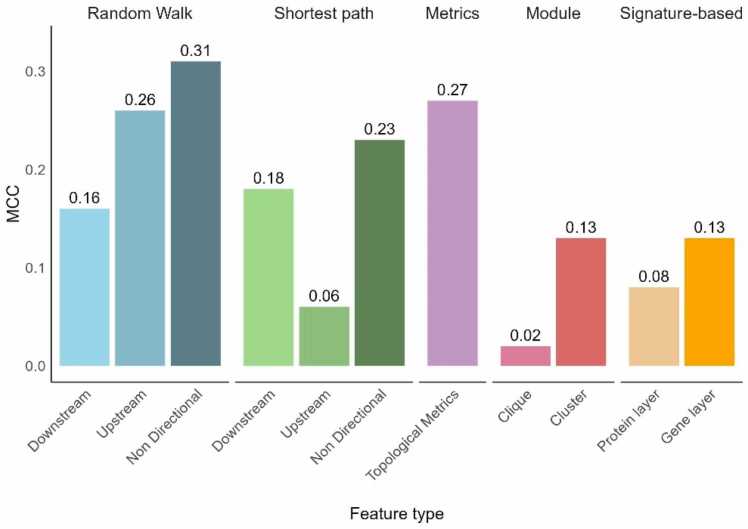
Barplot showing the performances of the KNN algorithm on the independent test set for each feature type using the Matthew Correlation Coefficient (MCC). An MCC equal to 0 represents a random classification, while an MCC equal to 1 represents a perfect classification. Here, Module-based, and Signature-based features had the lowest accuracies, compared to Propagation-based and Similarity-based features. The Random Walk method had overall better results than the Shortest Paths method.

To evaluate and compare the graph-based approaches selected in this study, the performance of the KNN algorithm in predicting therapeutic targets for each category of features was assessed using MCC on the independent test set. The graph-based methods evaluated here include Propagation-based methods (Random Walks with Restart and Shortest path on Downstream, Upstream or Non-directed interactions), Topological Metrics, Module-based methods (proximity to clusters or cliques), and Signature-based methods (proximity to genes or proteins associated with prostate cancer) (Fig. 3).

Module-based methods resulted in a lower MCCs, revealing that the clusters identified were not especially relevant to discriminate prostate cancer targets from other targets, and the KNN trained on clique-based features had performances comparable to random classification (Fig. 3). Surprisingly, Signature-based features did not lead to good classification performances either, despite their ubiquitous uses in target repositioning approaches (Average MCC=0.105) and were bested by Propagation-based approaches (Average MCC=0.20) and Topological Metrics (MCC=0.27). However, the results reveal that mapping prostate cancer signatures on the gene layer achieve better results than mapping them on the PPI network. This improvement further highlights the advantages of a multi-layer network compared to simple PPI networks for disease modelling. Additionally, Propagation-based features were quite predictive with random walks demonstrating superior performances in average compared to the shortest paths approach. The directionality of the network seems to greatly influence their results, but in opposite ways as *Upstream* propagation achieved good results using Random Walks (MCC = 0.21), but very poor results using Shortest Paths (MCC = 0.06), and inversely for *Downstream* propagation. Nevertheless, both methods obtained their maximal MCCs using non-directed interactions, results that were not anticipated and could reveal that non-directed propagation better grasp biological insights from complex multi-layer networks.

### 3.6 New targets discovery

A KNN algorithm was trained to predict novel targets for prostate cancer using an ensemble approach (Section 2.8). The features used were selected with variable selection (Section 2.6) from the two most predictive feature categories, Non-directed Random Walks and Topological Metrics. The algorithm identified a total of 10 therapeutic targets against prostate cancer that were not initially labelled as positive in our dataset (Table 3).

**Table 3:** Targets predicted with their known ligands. The indices specify the relation between the ligand and prostate cancer ranging from A to C with A: prescribed, B: gone though clinical trials, C: proposed in published literature.

Candidate	Molecular pathways	Biological functions	Known drugs	Previous indication
ADORA2	Adenosine Signaling Pathway	Regulates neurotransmitter release, and modulates inflammation	Adenosine, Caffeine (C) [85], Theophylline, Choline theophyllinate, Bufylline	Arrhythmias, Nervous system stimulant, Respiratory conditions
CSK	Regulation of Src Family Kinases	Involved in the control of cell proliferation, and immune response	Dasatinib (B) [86], Ibrutinib (B) [87] and Ponatinib	Leukemia
CYP24A1	Vitamin D Metabolism	Regulates calcium homeostasis, bone metabolism, and immune function	Ketoconazole (A) [88], Calcitriol (B) [89], Paricalcitol (B) [90]	Antifungal
BRDT	Epigenetic Regulation	Involved in spermatogenesis, chromatin remodeling, and transcriptional regulation	Birabresib (B) [91] and Fedratinib (C) [92]	Bone marrow cancer
BRAF	MAPK Signaling Pathway	Regulates cell growth, differentiation, and survival	Sorafenib (B) [93], Vemurafenib (B) [94] and Regorafenib (C) [95]	Solid tumors
IGF2R	Insulin-like Growth Factor Signaling	Involved in cell growth, differentiation, and apoptosis	Pabinafusp alfa	Metabolic diseases
C5AR	Complement System Activation	Mediates inflammation and immune response	Avacopan	Wegener's Granulomatosis
RAB7	Endocytic Pathway	Regulates vesicle trafficking and lysosomal biogenesis	No approved drug	
SETD2	Histone Methylation	Transcriptional regulation and DNA repair processes	No approved drug	
NPBWR1	Neuropeptide Signaling	Energy homeostasis and stress response	No approved drug	

Among these targets, five of them (A2AR/ADORA2A, CSK, CYP24A1, BRDT, and BRAF) have been suggested in recent scientific papers to be related to prostate cancer and all can interact with ligands with known therapeutic advantages against advanced prostate cancer. ADORA2A and CYP24A1 demonstrated therapeutic drug synergies in advanced prostate cancer when targeted in combination with existing treatments, PARP inhibitors [96] and calcitriol [97], respectively. On the other hand, CSK can interact with the known drug Dasatinib [86], and BRDT can interact with Birabresib [91], both drugs went into clinical trials for castration-resistant prostate cancer revealing their potential as therapeutic targets. BRAF, frequently mutated in prostate cancer is currently studied as a pan-cancer therapeutic target for solid tumors [98].

Additionally, four novel targets were predicted (IGF2R, C5AR/C5AR1, RAB7, and SETD2) which are all involved in molecular pathways closely connected to prostate cancer, with some having been recently proposed as suitable targets for other types of cancers. For example, C5AR expression was shown to promote prostate cancer proliferation and invasion and is involved in the immune response by being a receptor for the complement system, a key regulator of tumorigenesis that regulates inflammation in prostate cancer [99]. RAB7 is involved in vesicle transfer and lysosomal biogenesis and was shown to be involved in chemoresistance and a potential therapeutic target for colorectal cancer [100]. It was also shown to have a possible role in prostate cancer progression [101]. SETD2 is an enzyme involved in transcription elongation and splicing and its expression was correlated with prostate cancer survival. Its known inhibitors demonstrated the ability to inhibit proliferation in leukemia and multiple myeloma cells [102]. IGF2R is directly involved in the insulin-like growth factor axis, a pathway known to be important in prostate cancer and already proposed as a potential therapeutic avenue for prostate cancer [103].

Finally, the last predicted protein is a relatively unknown target that we suggest can be used as a novel potential treatment for prostate cancer. NPBWR1, also known as GPR7, has been associated with prostate cancer prognosis [104], but its role in tumor development and its potential use as a therapeutic target has yet to be understood. Because our prediction strategy relies on a biological network and extracted features that are transparent and interpretable, we can infer why NPBWR1 was chosen as a candidate protein and how targeting it would induce therapeutic effects. Based on its edges in the network, we know that it is involved in GCPR signaling and is activated by neuropeptides and opioid ligands. Opioid receptors have been heavily linked to prostate cancer biology [105], [106] and some have been proposed as potential targets such as OPRK1 [107] which hints at the possibility of NPBWR1 being able to be targeted in a similar way. Additionally, based on the topological similarities extracted in Section 2.5, two proteins stand out as the most like NPBWR1, GNAI1 and GNAI2. Indeed, NPBWR1 shares 96 % percent of its protein partners with both GNAI1 and GNAI2 (26 out of 27 protein interactions), revealing that they might act on similar molecular pathways within prostate cancer. Literature search reveals that GNAI1 is involved in prostate cancer growth [108] and GNAI2 is essential in prostate cancer cell migration [109] and invasion [110] and is also a crucial regulator in another hormone-dependent cancer, ovarian cancer [111]. These findings underscore the potential undiscovered role of NPBWR1 in prostate cancer, suggesting that targeting this protein with small molecules could induce a therapeutic response in prostate cancer.

These findings highlighted that the features extracted by non-directed random walks and local similarities with the KNN algorithm had effectively predicted targets closely associated with prostate cancer and its altered pathways. Some of these targets had expression values directly correlated with survival and prostate cancer progression, while others could interact with known molecules with therapeutic benefits.

## 4 Discussion

This work presents a novel approach integrating multi-layer network analysis with machine learning algorithms for repositioning therapeutic targets applied to prostate cancer. Our approach provided a performance-based comparison for different network-based approaches in target repositioning, revealing new insights into biological network analysis. These approaches included propagation-based methods (Random Walks with Restart and Shortest Paths), Topological Metrics, Module Identification and Signature-based methods which represented proximity to nodes associated with the disease.

The two best performing methods were non-directed Random Walks (MCC = 0.31) and Topological Metrics (which were mostly comprised of network-based similarities between two nodes using the number of shared neighboring nodes, MCC = 0.27), leading to the conclusion that both local structures, and global multi-layer network information were relevant for accurate prediction, which was in accordance with previously published research [19]. Random walks, which integrates the whole network’s structure during propagation also resulted in better performance compared to shortest paths, which might not be as suited to explore complex multi-layer networks. Both methods however were highly influenced by the type of propagation (*Downstream*, *Upstream* or *Non-directed*), with *Non-directed* propagation leading to the best classification accuracy. Additionally, by exploring the most predictive features identified through variable selection (Sections 3.2 and 3.3), we discovered that exploiting multiple types of disease-associated genes (such as mutation, CNAs, and DEGs) resulted in additional predictive features compared to exploiting DEGs alone. Even if proximity to DEGs accounted for most of the predictive information, which agrees with their frequent use in target repositioning. A similar result was observed with the additional network layers (gene, metabolite, GO, Molecular Pathway), the protein layer (PPI) was the most informative layer, but the other layers brought additional information that was also relevant for predicting prostate cancer therapeutic targets.

Despite the common use of topological metrics such as degree, betweenness, and centrality in target repositioning reported in the literature [12], [13], [14], [15], these metrics were not found predictive and did not pass the feature selection process in our case. This advocates for avoiding the use of generic topological metrics to predict the therapeutic properties of proteins. The most predictive Topological Metric was the network-based similarity with other nodes, which later yielded the second-best accuracy (MCC = 0.27). Moreover, proximities to prostate cancer signatures were not more predictive compared to other methods despite the ubiquitous use of disease signatures in target repositioning [16], [17], [18], [19]. Therapeutic targets for a disease are usually closer to known dysregulated genes or are themselves dysregulated [16], and this rationale is the support for many methods of target repositioning. This approach resulted in poorer performances in our case, with unbiased methods (Propagation-based and Topological metrics) having better performances. The reason for this lack of predictive power could be manyfold and potentially solved by developing better proximity measures to nodes associated with a disease. Ongoing efforts in network-based repositioning include considering biological pathways for improved proximity measures, which could better represent biological mechanisms and explain the effects of these alterations on prostate cancer biology, hence improving treatment discovery and target repositioning. Module-based methods (clusters or cliques) had limited accuracy as well in our comparison despite the various module identification algorithms utilized, revealing that module-based methods seemed not well suited to predict therapeutic potential in proteins in our case study. Future research focusing on different diseases and biological networks could give further insights into the usefulness of these approaches in target repositioning. To facilitate this research, an R package (github.com/MilanPicard/DiscoNet) was developed which integrates the methods exploited in this study. The package contains several functions to extract graph-based features for every node in any biological network, as well as parallelized variable selection methods to reduce the dimensionality of the resulting datasets.

However, supervised machine learning algorithms presented several limitations for target repositioning. The primary limitation is that supervised machine learning requires binary labeling (positive/negative) for each observation. However, the negative observations (negative targets) were mostly unlabelled observations rather than true negatives. This data setting in machine learning is known as positive-unlabelled learning (PU learning) and has already been used in target repositioning research [12], [21]. Several PU learning approaches exist, including modified classifiers, the identification of reliable negatives, and ensemble strategies [112]. Modified classifiers for PU learning are often less accessible and established than regular supervised classifiers, making it difficult to evaluate various types of machine learning algorithms on our data, which could hinder performances. Additionally, it is also difficult and even impossible in most cases to identify true negative proteins targets for prostate cancer. Therefore, we designed our approach based on the ensemble strategy, where unlabelled targets were considered negatives and the few positive targets expected among them were identified by training a classifier repeatedly on different bootstraps. Negatives targets repeatedly mislabelled as positive by the classifiers were considered “positive” candidates likely involved in therapeutic responses for prostate cancer. However, classifiers that predicted too many “positive” candidates could not be differentiated from classifiers making many mistakes. Thus, the outcome of this strategy is fewer repositioning predictions, as only classifier proposing few novel targets could be selected during training. In return, fewer target candidates were selected but with higher confidence, which was in line with our goal to propose only a handful of the most promising therapeutic targets for prostate cancer for further validation.

Finally, features extraction from the multi-layer network allowed the identification of the best features that were later used to train machine learning algorithms predicting new therapeutic targets for prostate cancer. The KNN algorithm, which showed the best classification performances, predicted ten proteins as therapeutic candidates (“positive candidates”). Compared to network-based drug repositioning that usually targets the same known proteins and therefore lead to treatment resistances, target repositioning circumvents this issue by predicting new proteins to target. The ten targets predicted in our case were all involved in diverse cellular activities such as calcium homeostasis, immune response, spermatogenesis, or neurotransmitter release. This diversity of biological functions suggests involvement of these targets in various biological pathways that could be related to prostate cancer, thus increasing the probabilities of finding new treatments not impacted by known resistance mechanisms. By inhibiting the tumor in different ways, it also promotes the discovery of drug synergies. For example, two predicted targets out of ten, ADORA2A and CYP24A1, had already known benefits when targeted in combination with established treatments (PARP inhibitors [96] and calcitriol [97], respectively), showing the ability of target repositioning to discover drug combinations.

Among the 10 targets predicted, five were currently under investigation. The others, while never having been pursued as therapeutic target candidates for prostate cancer, were found after literature review to be all involved in biological processes strongly associated with the disease development and progression (Section 3.6), which may warrant further investigation. However, our repositioning approach also discovered NPBWR1, a relatively unknown protein which therapeutic potential could not be validated by literature review only. Here, we showcased the importance of using interpretable features to train machine learning algorithms to shed light on these novel predictions (Section 3.6). Topological Metrics for example, which were utilized for the prediction of NPBWR1, represented network-based similarities between nodes. Using these similarities, we discovered that NPBWR1 was highly similar to two other proteins in the network, GNAI1 involved in prostate cancer growth [108] and GNAI2 involved in prostate cancer cell migration [109] and invasion [110]. Through network analysis and interpretable features, the prediction of NPBWR1 could therefore be explained, which could not have been done using feature embedding methods, which derived features using weight optimization by neural networks models and are therefore difficult to interpret. The inclusion of interpretable features was a deliberate choice to anchor novel targets in biological understanding. These results highlighted the benefits of the developed approach for target repositioning based on network analysis.

## 5 Conclusion

This study exploited a large multi-layer network and diverse network-based approaches to repurpose therapeutic targets for prostate cancer. Using machine learning algorithms, the effectiveness of these various approaches was systematically assessed and key insights into network analysis were presented. We showed the benefits of additional biological data (multiple network layers) as opposed to relying solely on PPI networks, and the importance of local topological similarities and global propagation methods to accurately predict therapeutic targets. We also revealed that commonly used approaches such as module identification and proximity to disease-associated nodes performed surprisingly poorly when applied in our case and should not be used systematically. Using our approach, ten diverse protein targets were identified. Five of them represent new and untested candidates which all showed promising avenues to combat drug resistance in advanced prostate cancer. This study provided a robust framework for advancing our understanding of complex networks and their use in disease and treatment discovery.

## Funding

This work was supported by the Research and Innovation chair L′Oréal in Digital Biology.

## Author statement

MP wrote the manuscript and designed the figures. MP, ML, MPSB, AB, JP, and OP revised the manuscript. AD supervised research.

## CRediT authorship contribution statement

**Milan Picard:** Writing – original draft. **Marie-Pier Scott-Boyer:** Writing – review & editing. **Arnaud Droit:** Supervision. **Antoine Bodein:** Writing – review & editing. **Mickaël Leclercq:** Writing – review & editing. **Julien Prunier:** Writing – review & editing. **Olivier Périn:** Writing – review & editing.

## Declaration of Competing Interest

The authors declare that they have no known competing financial interests or personal relationships that could have appeared to influence the work reported in this paper.

## References

[1] Jarada T.N., Rokne J.G., Alhajj R. (2020). A review of computational drug repositioning: strategies, approaches, opportunities, challenges, and directions. J Chemin-.

[2] Jourdan J.P., Bureau R., Rochais C., Dallemagne P. (2020). Drug repositioning: a brief overview. J Pharm Pharmacol.

[3] Xue H., Li J., Xie H., Wang Y. (2018). Review of drug repositioning approaches and resources. Int J Biol Sci.

[4] Parisi D. (2020). Drug repositioning or target repositioning: a structural perspective of drug-target-indication relationship for available repurposed drugs. Comput Struct Biotechnol J.

[5] Sun D., Gao W., Hu H., Zhou S. (2022). Why 90% of clinical drug development fails and how to improve it?. Acta Pharm Sin B.

[6] Park K. (2019). A review of computational drug repurposing. Transl Clin Pharmacol.

[7] Lin A. (2019). Off-target toxicity is a common mechanism of action of cancer drugs undergoing clinical trials. Sci Transl Med.

[8] Sun W., Sanderson P.E., Zheng W. (2016). Drug combination therapy increases successful drug repositioning. Drug Discov Today.

[9] Housman G. (2014). Drug resistance in cancer: an overview. Cancers.

[10] Picard M., Scott-Boyer M.P., Bodein A., Périn O., Droit A. (2021). Integration strategies of multi-omics data for machine learning analysis. Comput Struct Biotechnol J.

[11] Robin V. (2022). Overview of methods for characterization and visualization of a protein–protein interaction network in a multi-omics integration context. Front Mol Biosci.

[12] Dezso Z., Ceccarelli M. (2020). Machine learning prediction of oncology drug targets based on protein and network properties. BMC Bioinforma.

[13] Podder A., Pandit M., Narayanan L. (2018). Drug target prioritization for Alzheimer’s disease using protein interaction network analysis. OMICS.

[14] Amala A., Emerson I.A. (2019). Identification of target genes in cancer diseases using protein–protein interaction networks.. Netw Model Anal Health Inform Bioinforma.

[15] Bidkhori G. (2018). Metabolic network-based identification and prioritization of anticancer targets based on expression data in hepatocellular carcinoma. Front Physiol.

[16] Isik Z., Baldow C., Cannistraci C.V., Schroeder M. (2015). Drug target prioritization by perturbed gene expression and network information. Sci Rep.

[17] Han S., Hong J., Yun S.J., Koo H.J., Kim T.Y. (2023). PWN: enhanced random walk on a warped network for disease target prioritization. BMC Bioinforma.

[18] Yang J. (2021). Network-based target prioritization and drug candidate identification for multiple sclerosis: from analyzing ‘omics data’ to druggability simulation. ACS Chem Neurosci.

[19] Emig D. (2013). Drug target prediction and repositioning using an integrated network-based approach. PLoS One.

[20] Tsuji S. (2021). Artificial intelligence-based computational framework for drug-target prioritization and inference of novel repositionable drugs for Alzheimer’s disease. Alzheimers Res Ther.

[21] Muslu O., Hoyt C.T., Lacerda M., Hofmann-Apitius M., Frohlich H. (2022). GuiltyTargets: prioritization of novel therapeutic targets with network representation learning. IEEE/ACM Trans Comput Biol Bioinform.

[22] Kotlyar M., Fortney K., Jurisica I. (2012). Network-based characterization of drug-regulated genes, drug targets, and toxicity. Methods.

[23] Lucchetta M., List M., Blumenthal D.B., Schaefer M.H. (2023). Emergence of power-law distributions in protein-protein interaction networks through study bias. bioRxiv.

[24] Lazareva O., Baumbach J., List M., Blumenthal D.B. (2021). On the limits of active module identification. Brief Bioinforma.

[25] Schaefer M.H., Serrano L., Andrade-Navarro M.A. (2015). Correcting for the study bias associated with protein-protein interaction measurements reveals differences between protein degree distributions from different cancer types. Front Genet.

[26] Baptista A., Gonzalez A., Baudot A. (2022). Universal multilayer network exploration by random walk with restart. Commun Phys.

[27] Liu X. (2020). Robustness and lethality in multilayer biological molecular networks. Nat Commun.

[28] Li Z.C. (2015). Large-scale identification of potential drug targets based on the topological features of human protein-protein interaction network. Anal Chim Acta.

[29] Chen H.G., Zhou X.H. (2021). Mnbdr: a module network based method for drug repositioning. Genes.

[30] Wang R.S., Loscalzo J. (2021). Network module-based drug repositioning for pulmonary arterial hypertension. CPT Pharmacomet Syst Pharm.

[31] Sadegh S. (2021). Network medicine for disease module identification and drug repurposing with the NeDRex platform. Nat Commun.

[32] Luo H. (2019). Computational drug repositioning with random walk on a heterogeneous network. IEEE/ACM Trans Comput Biol Bioinform.

[33] Wang Y., Guo M., Ren Y., Jia L., Yu G. (2019). Drug repositioning based on individual bi-random walks on a heterogeneous network. BMC Bioinforma.

[34] Cheng X. (2022). Drug repurposing for cancer treatment through global propagation with a greedy algorithm in a multilayer network. Cancer Biol Med.

[35] Fahimian G., Zahiri J., Arab S.S., Sajedi R.H. (2020). RepCOOL: computational drug repositioning via integrating heterogeneous biological networks. J Transl Med.

[36] Aydin B., Beklen H., Arga K.Y., Bayrakli F., Turanli B. (2023). Epigenomic and transcriptomic landscaping unraveled candidate repositioned therapeutics for non-functioning pituitary neuroendocrine tumors. J Endocrinol Invest.

[37] Mokou M. (2023). A drug repurposing pipeline based on bladder cancer integrated proteotranscriptomics signatures. Methods Mol Biol Vol 2684.

[38] Daminelli S., Haupt V.J., Reimann M., Schroeder M. (2012). Drug repositioning through incomplete bi-cliques in an integrated drug-target-disease network. Integr Biol.

[39] Pernar, C.H., Ebot, E.M., Wilson, K.M. & Mucci, L.A. The Epidemiology of Prostate Cancer; 2018. 10.1101/cshperspect.a030361. PMC6280714 29311132

[40] Schröder F., Crawford E.D., Axcrona K., Payne H., Keane T.E. (2012). Androgen deprivation therapy: past, present and future. BJU Int.

[41] Weng H. (2017). Androgen receptor gene polymorphisms and risk of prostate cancer: a meta-analysis. Sci Rep.

[42] Gregory C.W., Johnson R.T., Mohler J.L., French F.S., Wilson E.M. (2001). Androgen receptor stabilization in recurrent prostate cancer is associated with hypersensitivity to low androgen. Cancer Res.

[43] Bahmad H.F. (2022). Overcoming drug resistance in advanced prostate cancer by drug repurposing. Med Sci.

[44] Powell K. (2015). ERG/AKR1C3/AR constitutes a feed-forward loop for AR signaling in prostate cancer cells. Clin Cancer Res.

[45] Wishart D.S. (2018). DrugBank 5.0: a major update to the drugbank database for 2018. Nucleic Acids Res.

[46] Chen X., Ji Z.L., Chen Y.Z. (2002). TTD: therapeutic target database. Nucleic Acids Res.

[47] Carlson M., Falcon S., Pages H., Li N. (2019). org. Hs. eg. db: genome wide annotation for Human. R Package Version.

[48] Luck K. (2020). A reference map of the human binary protein interactome. Nature.

[49] Szklarczyk D. (2015). STRING v10: protein-protein interaction networks, integrated over the tree of life. Nucleic Acids Res.

[50] Alonso-López Di (2019). APID database: redefining protein-protein interaction experimental evidences and binary interactomes. Database.

[51] Patil A., Nakai K., Nakamura H. (2011). HitPredict: a database of quality assessed protein-protein interactions in nine species. Nucleic Acids Res.

[52] Huang H.Y. (2022). MiRTarBase update 2022: an informative resource for experimentally validated miRNA-target interactions. Nucleic Acids Res.

[53] Sticht C., De La Torre C., Parveen A., Gretz N. (2018). Mirwalk: an online resource for prediction of microrna binding sites. PLoS One.

[54] Chi S.M. (2014). REGNET: mining context-specific human transcription networks using composite genomic information. BMC Genom.

[55] Bovolenta L.A., Acencio M.L., Lemke N. (2012). HTRIdb: an open-access database for experimentally verified human transcriptional regulation interactions. BMC Genom.

[56] Han H. (2018). TRRUST v2: an expanded reference database of human and mouse transcriptional regulatory interactions. Nucleic Acids Res.

[57] Garcia-Alonso L., Holland C.H., Ibrahim M.M., Turei D., Saez-Rodriguez J. (2019). Benchmark and integration of resources for the estimation of human transcription factor activities. Genome Res.

[58] Tong Z., Cui Q., Wang J., Zhou Y. (2019). TransmiR v2.0: an updated transcription factor-microRNA regulation database. Nucleic Acids Res.

[59] Kanehisa M., Goto S. (2000). KEGG: kyoto encyclopedia of genes and genomes. Nucleic Acids Res.

[60] Croft D. (2011). Reactome: a database of reactions, pathways and biological processes. Nucleic Acids Res.

[61] Slenter D.N. (2018). WikiPathways: a multifaceted pathway database bridging metabolomics to other omics research. Nucleic Acids Res.

[62] Sales G., Calura E., Cavalieri D., Romualdi C. (2012). Graphite - a bioconductor package to convert pathway topology to gene network. BMC Bioinforma.

[63] Grote, S. Title Gene ontology enrichment using FUNC. R package version *1.18.0* R package; 2022.

[64] Thul P.J., Lindskog C. (2018). The human protein atlas: a spatial map of the human proteome. Protein Sci.

[65] Lachmann A. (2018). Massive mining of publicly available RNA-seq data from human and mouse. Nat Commun.

[66] Cerami E. (2012). The cBio cancer genomics portal: an open platform for exploring multidimensional cancer genomics data. Cancer Discov.

[67] Li, R., Zhu, J., Zhong, W.-D. & Jia, Z. PCaDB - a comprehensive and interactive database for transcriptomes from prostate cancer population cohorts. *bioRxiv;* 2022.

[68] Csardi G., Nepusz T. (2006). The igraph software package for complex network research. Inter Complex Syst.

[69] Valdeolivas A. (2019). Random walk with restart on multiplex and heterogeneous biological networks. Bioinformatics.

[70] Choobdar S. (2019). Assessment of network module identification across complex diseases. Nat Methods.

[71] Tomasoni M. (2020). MONET: a toolbox integrating top-performing methods for network modularization. Bioinformatics.

[72] Guney E., Menche J., Vidal M., Barábasi A.L. (2016). Network-based in silico drug efficacy screening. Nat Commun.

[73] Sheils T.K. (2021). TCRD and pharos 2021: mining the human proteome for disease biology. Nucleic Acids Res.

[74] Gaulton A. (2012). ChEMBL: a large-scale bioactivity database for drug discovery. Nucleic Acids Res.

[75] Azhagusundari B., Thanamani A.S. (2013). Feature selection based on information gain. Int J Innov Technol Explor Eng.

[76] Hornik K., Buchta C., Zeileis A. (2009). Open-source machine learning: R meets weka. Comput Stat.

[77] Kuhn M. (2008). Building predictive models in R using the caret package. J Stat Softw.

[78] Friedman J., Hastie T., Tibshirani R. (2010). Regularization paths for generalized linear models via coordinate descent. J Stat Softw.

[79] Liaw A., Wiener M. (2002). Classification and regression by randomforest. R N.

[80] Meyer D. (2022). e1071: misc functions of the department of statistics, probability theory group (Formerly: E1071), TU Wien. R Package Version 1 7-12.

[81] LeDell, E. et al. R Interface for the ‘H2O’ scalable machine learning platform; 2020.

[82] Zhou M. (2022). Patterns of structural variation define prostate cancer across disease states. JCI Insight.

[83] Giotti B. (2019). Assembly of a parts list of the human mitotic cell cycle machinery. J Mol Cell Biol.

[84] Chicco D., Jurman G. (2023). The matthews correlation coefficient (MCC) should replace the ROC AUC as the standard metric for assessing binary classification. BioData Min.

[85] Chen X., Zhao Y., Tao Z., Wang K. (2021). Coffee consumption and risk of prostate cancer: a systematic review and meta-analysis. BMJ Open.

[86] Twardowski P.W. (2013). A phase II trial of dasatinib in patients with metastatic castration-resistant prostate cancer treated previously with chemotherapy. Anticancer Drugs.

[87] Zhu Z., Ling L., Qi L., Chong Y., Xue L. (2020). Bruton’s tyrosine kinase (BTK) inhibitor (ibrutinib)-suppressed migration and invasion of prostate cancer. Onco Targets Ther.

[88] Patel V., Liaw B., Oh W. (2018). The role of ketoconazole in current prostate cancer care. Nat Rev Urol.

[89] Ben-Eltriki M., Deb S., Tomlinson Guns E.S. (2016). Calcitriol in combination therapy for prostate cancer: Pharmacokinetic and pharmacodynamic interactions. J Cancer.

[90] Schwartz G.G. (2005). Phase I/II study of 19-nor-1α-25-dihydroxyvitamin D2 (paricalcitol) in advanced, androgen-insensitive prostate cancer. Clin Cancer Res.

[91] Lewin J. (2018). Phase Ib trial with birabresib, a small-molecule inhibitor of bromodomain and extraterminal proteins, in patients with selected advanced solid tumors. *J Clin Oncol*.

[92] Nalairndran G. (2021). Inhibition of Janus Kinase 1 synergizes docetaxel sensitivity in prostate cancer cells. J Cell Mol Med.

[93] Dahut W.L. (2008). A phase II clinical trial of sorafenib in androgen-independent prostate cancer. Clin Cancer Res.

[94] Wozniak D.J. (2019). Vemurafenib inhibits active PTK6 in PTEN-null prostate tumor cells. Mol Cancer Ther.

[95] Mirantes C. (2016). Effects of the multikinase inhibitors Sorafenib and Regorafenib in PTEN deficient neoplasias. Eur J Cancer.

[96] McCann P.J. (2023). Abstract B032: combined A2AR and PARP inhibition in homologous recombination deficient (HRD) castrate-resistant prostate cancer (CRPC). Cancer Res.

[97] Muindi J.R. (2010). CYP24A1 inhibition enhances the antitumor activity of calcitriol. Endocrinology.

[98] Chehrazi-Raffle A. (2023). Unique spectrum of activating BRAF alterations in prostate cancer. Clin Cancer Res.

[99] Netti G.S. (2021). Role of complement in regulating inflammation processes in renal and prostate cancers. Cells.

[100] Guerra F., Bucci C. (2019). Role of the RAB7 protein in tumor progression and cisplatin chemoresistance. Cancers.

[101] Steffan J.J. (2014). Supporting a role for the GTPase rab7 in prostate cancer progression. PLoS One.

[102] Takahashi S., Takada I. (2023). Recent advances in prostate cancer: WNT signaling, chromatin regulation, and transcriptional coregulators. Asian J Androl.

[103] Heidegger I., Massoner P., Sampson N., Klocker H. (2015). The insulin-like growth factor (IGF) axis as an anticancer target in prostate cancer. Cancer Lett.

[104] Cottrell S. (2007). Discovery and validation of 3 Novel DNA methylation markers of prostate cancer prognosis. J Urol.

[105] Lec P.M. (2020). The role of opioids and their receptors in urological malignancy: a review. J Urol.

[106] Yamashita H., Shuman L., Warrick J.I., Raman J.D., Degraff D.J. (2018). Androgen represses opioid growth factor receptor (OGFR) in human prostate cancer LNCaP cells and OGFR expression in human prostate cancer tissue. Am J Clin Exp Urol.

[107] Makino Y. (2022). Comprehensive genomics in androgen receptor-dependent castration-resistant prostate cancer identifies an adaptation pathway mediated by opioid receptor kappa 1. Commun Biol.

[108] Lau K.M. (2017). Activation of GPR30 stimulates GTP-binding of Gαi1 protein to sustain activation of Erk1/2 in inhibition of prostate cancer cell growth and modulates metastatic properties. Exp Cell Res.

[109] Zhong M., Clarke S., Vo B.H.T., Khan S.A. (2012). The essential role of Giα2 in prostate cancer cell migration.. Mol Cancer Res.

[110] Caggia S. (2018). Novel role of Giα2 in cell migration: downstream of PI3-kinase–AKT and Rac1 in prostate cancer cells. J Cell Physiol.

[111] Raymond J.R., Appleton K.M., Pierce J.Y., Peterson Y.K. (2014). Suppression of GNAI2 message in ovarian cancer. J Ovarian Res.

[112] Li F. (2022). Positive-unlabeled learning in bioinformatics and computational biology: a brief review. Brief Bioinform.

