# Supplementary material for: Target repositioning using multi-layer networks and machine learning: The case of prostate cancer
Source: Comput Struct Biotechnol J. 2024 Jun 15;24:464–75. doi: 10.1016/j.csbj.2024.06.012 (PMC11231507; doi:10.1016/j.csbj.2024.06.012)
Supplement: Supplementary file 1 — Supplementary material [file mmc1.docx]

Supplementary data

Target repositioning using multi-layer networks and machine learning: the case of prostate cancer.

**
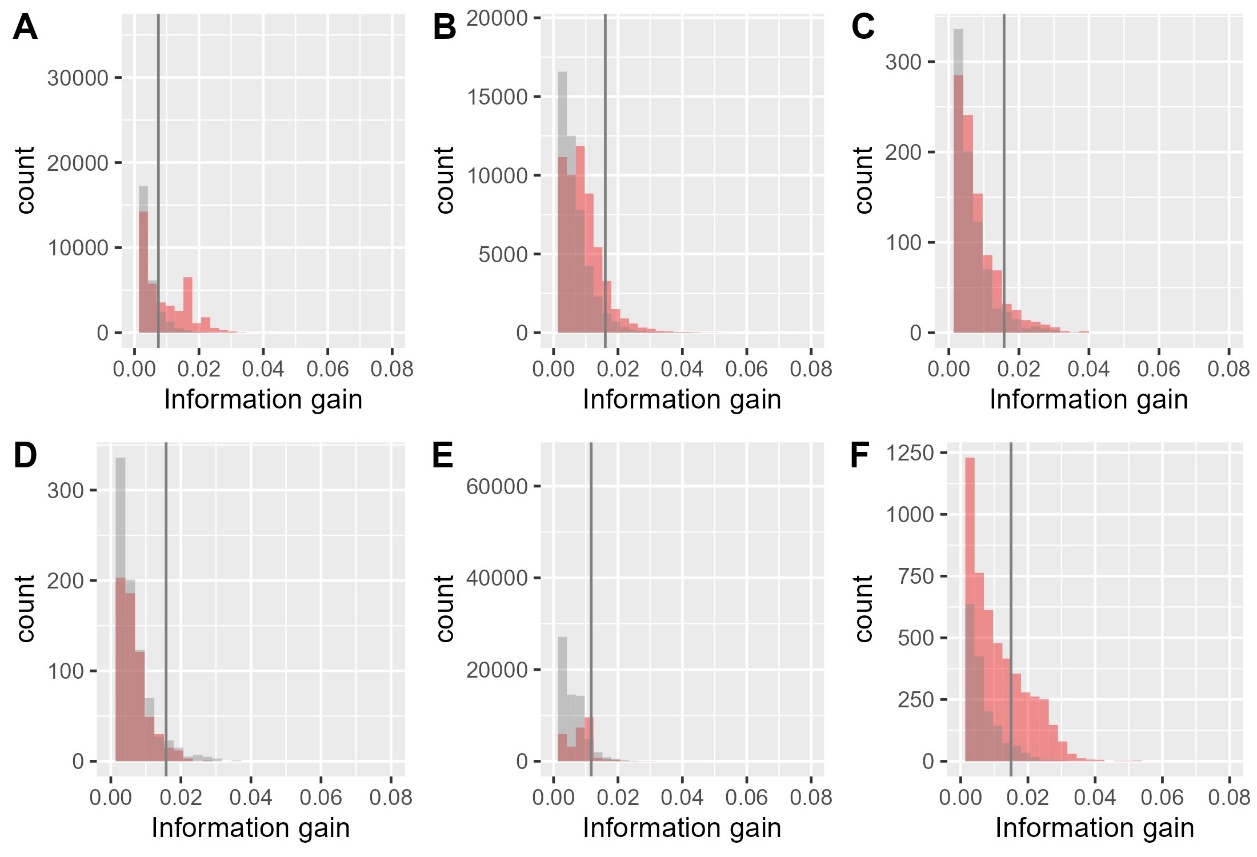
S1: Feature selection : information gain**

Figure S1: Distribution of information gain values for regular features (red) and for random features (gray). The random features were extracted using the same graph-based methods but using either random networks created by shuffling the edges or the original network, or by creating modules, cliques, or gene signature from randomly sampling nodes. These distributions show a large overlap between the information gain distributions, demonstrating that a lot of the regular features extracted are noisy and have discriminative power similar to random features. In order to remove noisy features, a cut-off based on the upper bound on Tukey's fences method for outlier detection was applied to each random distribution, as represented by the vertical line. Every feature with a higher information gain than this cut-off was therefore deemed to be relevant and kept at this step. The value of each cut-off is respectively, **A**: shortest paths, **B**: Random Walks, **C**: Topological similarity, **D**: Cluster, **E**: Clique, **F**: Signature-based.

**S2: Number of features selected after each step.**

| Dataset | Original | InfGain | Cor | RF | LASSO | Intersect | Union |
| --- | --- | --- | --- | --- | --- | --- | --- |
| RWR down | 65430 | 5329 | 2968 | 499 | 20 | 13 | 506 |
| RWR up | 65430 | 8264 | 5928 | 418 | 27 | 11 | 434 |
| RWR non | 65430 | 9693 | 7061 | 867 | 28 | 11 | 325 |
| SHP down | 63571 | 16966 | 3643 | 416 | 32 | 26 | 422 |
| SHP up | 62030 | 6037 | 3998 | 399 | 32 | 20 | 411 |
| SHP non | 65430 | 14891 | 7249 | 355 | 18 | 14 | 359 |
| Topo | 65439 | 1642 | 858 | 278 | 0 | 0 | 278 |
| Clique | 724 | 29 | 13 | 13 | 1 | 1 | 13 |
| Cluster | 1204 | 91 | 74 | 71 | 8 | 6 | 73 |
| Sign prot | 2227 | 97 | 43 | 35 | 1 | 1 | 35 |
| Sign Gene | 3917 | 1205 | 281 | 138 | 11 | 9 | 140 |

Table S2: Number of features extracted for each feature type across the different variable selection steps. From “Original” which is the initial number of features extracted from the network, variable selection methods used are in order: Information Gain threshold (InfGain), Correlation threshold (Cor), Random Forest (RF) importance measure threshold, Adaptive LASSO (LASSO) selection. The final set of features was the union set between RF and LASSO. The Intersect set was also given.

**S3: Hyperparameter tuning.**

The following hyperparameters were tuned. Weighted Support-Vector Machines: cost, class.weights, and gamma. Naïve Bayes: kernel, usekernel. Weighted k-nearest neighbors: kernel, ks, distance. Artificial Neural Networks: input_dropout_ratio, class_sampling_factors, hidden_dropout_ratio, mini_batch_size, hidden.


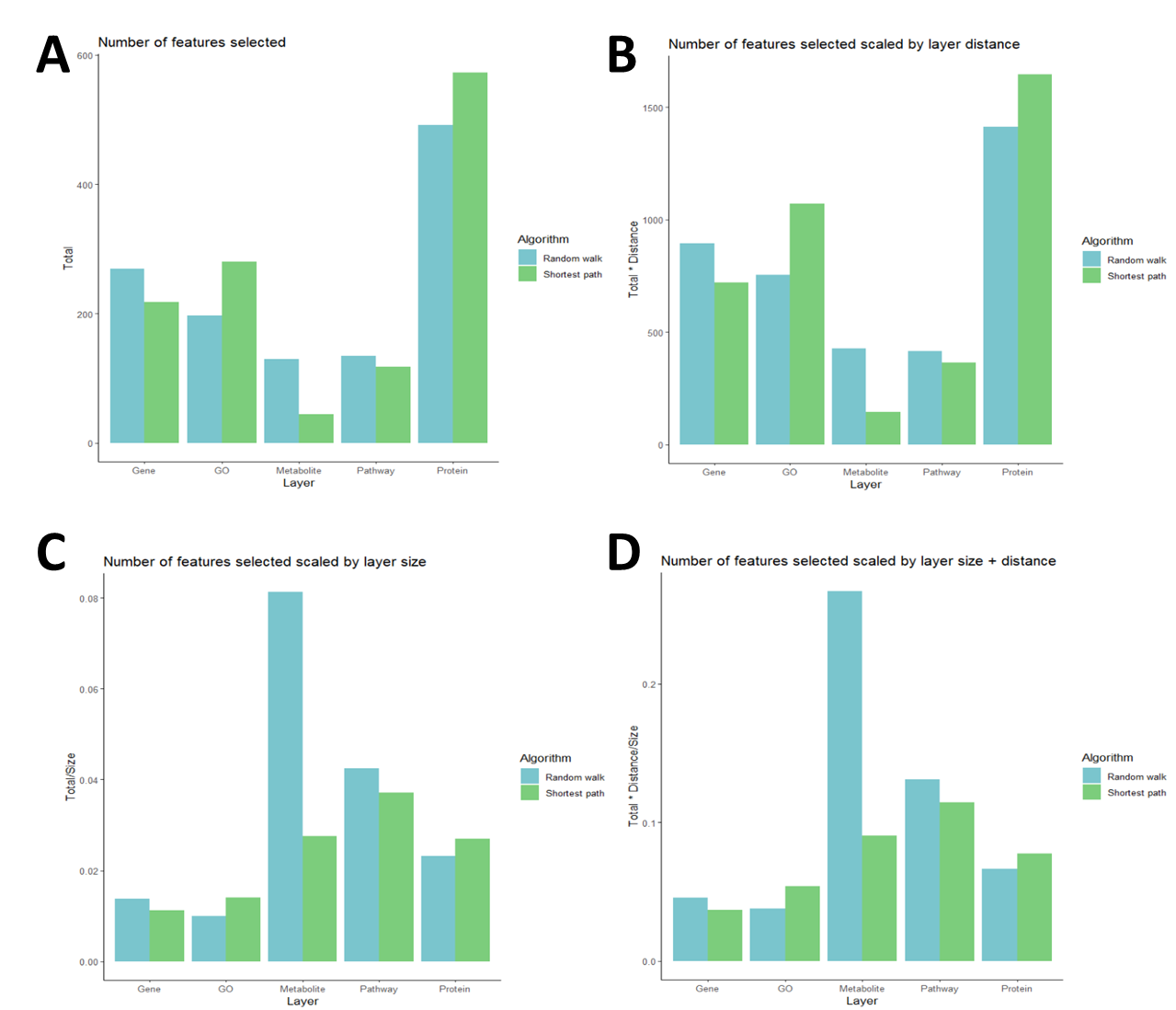
**S4: Layer importance by different parameters:**

Figure S4: The barplots show the numbers of propagation-based feature chosen. **A**: the original plot as shown in the article. **B**: The same data as A but normalized by the average distance between the layer considered and all targets. **C**: The same data as A but normalized by the node size of each layer. **D**: Normalized but node size and distance for each layer. These plots shows that layer proximity to targets do not really influence the relative importance between layers, and therefore the PPI layer is not biased and overly selected compared to other layers of similar size like the Gene layer. However, when taking layer size into account, the metabolite and pathway layer become the most important relative to the other ones, showing that even though they are smaller layers, they still contribute to bringing relevant information into the network.

**S5: Overlap between prostate cancer signatures and the network layers**

| Overlap | Mutated genes | Structural variants genes | CNA genes | DEG (+) | DEG (-) |
| --- | --- | --- | --- | --- | --- |
| Full Signature | 56 | 24 | 108 | 266 | 1466 |
| Overlap (TF's genes) | 36 | 18 | 33 | 120 | 811 |
| Overlap (miRNA's genes) | 39 | 16 | 32 | 104 | 655 |
| Overlap (PPI) | 54 | 18 | 43 | 174 | 1133 |
| Overlap (Full Network) | 53 | 21 | 49 | 181 | 1171 |

Table S5: Overlap between the prostate cancer signatures retrieved and the different layers of the network including the genes regulated by transcription factors (TF), the genes regulated by miRNAs, and the genes whose protein products interact within the PPI network.

**S6: Detailed metrics on training and test data for all feature types and models.**

Based on TN (*true negatives*), FN (*false negatives*), TP (*true positives*), FP (*false positives*), these metrics include:

- Specificity: TN/(TN+FP), number of correct negative predictions.
- Precision: TP/(TP+FN), number of correct positive predictions.
- Recall: TP/(TP+FP), number of positive observations correctly predicted.
- Balanced Accuracy: (Specificity + Precision)/2, average of specificity and precision.
- AUC: area under the Receiver Operating Characteristic (ROC) curve, which plots the Recall against the false positive rate (1-Specificity).
- MCC: (TPxTN-FPxFN)/√((TP+FP)x(TP+FN)x(TN+FP)x(TN+FN)), Matthews Correlation Coefficient.

**On the training data:**

- **Random Forest:**

| Dataset | Specificity | Precision | Recall | Balanced.Accuracy | AUC | MCC |
| --- | --- | --- | --- | --- | --- | --- |
| RWR down | 0.98 | 0.53 | 0.16 | 0.57 | 0.57 | 0.23 |
| RWR up | 0.98 | 0.59 | 0.19 | 0.58 | 0.58 | 0.28 |
| **RWR non** | **0.98** | **0.6** | **0.19** | **0.59** | **0.59** | **0.29** |
| SHP down | 0.98 | 0.52 | 0.18 | 0.58 | 0.58 | 0.25 |
| **SHP up** | **0.98** | **0.66** | **0.18** | **0.58** | **0.58** | **0.29** |
| SHP non | 0.98 | 0.58 | 0.17 | 0.57 | 0.57 | 0.26 |
| Topo | 0.97 | 0.49 | 0.13 | 0.55 | 0.55 | 0.19 |
| Clique | 0.97 | 0.42 | 0.1 | 0.53 | 0.53 | 0.13 |
| Cluster | 0.97 | 0.35 | 0.08 | 0.52 | 0.52 | 0.09 |
| Sign prot | 0.97 | 0.45 | 0.11 | 0.54 | 0.54 | 0.15 |
| Sign Gene | 0.97 | 0.44 | 0.12 | 0.54 | 0.54 | 0.16 |
| All feature | 0.98 | 0.59 | 0.18 | 0.58 | 0.58 | 0.27 |

- **SVM:**

| Dataset | Specificity | Precision | Recall | Balanced.Accuracy | AUC | MCC |
| --- | --- | --- | --- | --- | --- | --- |
| RWR down | 0.97 | 0.37 | 0.33 | 0.65 | 0.65 | 0.31 |
| RWR up | 0.97 | 0.41 | 0.48 | 0.73 | 0.73 | 0.41 |
| RWR non | 0.97 | 0.44 | 0.44 | 0.7 | 0.7 | 0.41 |
| SHP down | 0.98 | 0.39 | 0.24 | 0.61 | 0.61 | 0.28 |
| **SHP up** | **0.97** | **0.51** | **0.53** | **0.75** | **0.75** | **0.49** |
| SHP non | 0.96 | 0.34 | 0.42 | 0.69 | 0.69 | 0.34 |
| Topo | 0.98 | 0.39 | 0.25 | 0.61 | 0.61 | 0.28 |
| Clique | 0.93 | 0.07 | 0.11 | 0.52 | 0.52 | 0.03 |
| Cluster | 0.98 | 0.21 | 0.11 | 0.55 | 0.55 | 0.12 |
| Sign prot | 0.98 | 0.24 | 0.1 | 0.54 | 0.54 | 0.13 |
| Sign Gene | 0.93 | 0.12 | 0.22 | 0.57 | 0.57 | 0.11 |
| All feature | 0.98 | 0.52 | 0.36 | 0.67 | 0.67 | 0.4 |

- **Naïve Bayes:**

| Dataset | Specificity | Precision | Recall | Balanced.Accuracy | AUC | MCC |
| --- | --- | --- | --- | --- | --- | --- |
| RWR down | 0.94 | 0.22 | 0.37 | 0.65 | 0.65 | 0.24 |
| **RWR up** | **0.83** | **0.17** | **0.73** | **0.78** | **0.78** | **0.29** |
| RWR non | 0.83 | 0.16 | 0.69 | 0.76 | 0.76 | 0.27 |
| SHP down | 0.74 | 0.1 | 0.67 | 0.7 | 0.7 | 0.18 |
| SHP up | 0.77 | 0.13 | 0.76 | 0.76 | 0.76 | 0.25 |
| SHP non | 0.78 | 0.13 | 0.73 | 0.76 | 0.76 | 0.25 |
| Topo | 0.86 | 0.15 | 0.54 | 0.7 | 0.7 | 0.22 |
| Clique | 0.98 | 0.09 | 0.04 | 0.51 | 0.51 | 0.03 |
| Cluster | 0.93 | 0.13 | 0.21 | 0.57 | 0.57 | 0.11 |
| Sign prot | 0.98 | 0.28 | 0.09 | 0.54 | 0.54 | 0.12 |
| Sign Gene | 0.93 | 0.16 | 0.27 | 0.6 | 0.6 | 0.16 |
| All feature | 0.84 | 0.18 | 0.74 | 0.79 | 0.79 | 0.31 |

- **K-nearest neighbors:**

| Dataset | Specificity | Precision | Recall | Balanced.Accuracy | AUC | MCC |
| --- | --- | --- | --- | --- | --- | --- |
| RWR down | 0.98 | 0.34 | 0.2 | 0.59 | 0.59 | 0.23 |
| **RWR up** | **0.99** | **0.44** | **0.22** | **0.6** | **0.6** | **0.28** |
| RWR non | 0.99 | 0.4 | 0.19 | 0.59 | 0.59 | 0.24 |
| SHP down | 0.98 | 0.3 | 0.14 | 0.56 | 0.56 | 0.18 |
| SHP up | 1 | 0.55 | 0.12 | 0.56 | 0.56 | 0.22 |
| SHP non | 1 | 0.54 | 0.12 | 0.56 | 0.56 | 0.22 |
| Topo | 0.98 | 0.34 | 0.2 | 0.59 | 0.59 | 0.23 |
| Clique | 0.99 | 0.16 | 0.03 | 0.51 | 0.51 | 0.04 |
| Cluster | 0.99 | 0.25 | 0.06 | 0.53 | 0.53 | 0.1 |
| Sign prot | 0.97 | 0.14 | 0.11 | 0.54 | 0.54 | 0.09 |
| Sign Gene | 0.97 | 0.2 | 0.19 | 0.58 | 0.58 | 0.16 |
| All feature | 0.99 | 0.4 | 0.18 | 0.58 | 0.58 | 0.24 |

- **Neural Network**

| Dataset | Specificity | Precision | Recall | AUC | Balanced Accuracy | MCC |
| --- | --- | --- | --- | --- | --- | --- |
| RWR down | 0.91 | 0.32 | 0.61 | 0.85 | 0.61 | 0.38 |
| RWR up | 0.96 | 0.46 | 0.57 | 0.89 | 0.57 | 0.48 |
| RWR non | 0.97 | 0.62 | 0.57 | 0.9 | 0.57 | 0.54 |
| SHP down | 0.95 | 0.38 | 0.56 | 0.83 | 0.56 | 0.41 |
| **SHP up** | **0.98** | **0.71** | **0.53** | **0.9** | **0.53** | **0.56** |
| SHP non | 0.97 | 0.44 | 0.54 | 0.85 | 0.54 | 0.45 |
| Topo | 0.95 | 0.43 | 0.37 | 0.79 | 0.37 | 0.32 |
| Clique | 0.69 | 0.1 | 0.52 | 0.56 | 0.52 | 0.12 |
| Cluster | 0.9 | 0.35 | 0.41 | 0.69 | 0.41 | 0.28 |
| Sign prot | 0.88 | 0.36 | 0.44 | 0.7 | 0.44 | 0.27 |
| Sign Gene | 0.85 | 0.19 | 0.52 | 0.71 | 0.52 | 0.24 |
| All feature | 0.97 | 0.57 | 0.6 | 0.91 | 0.6 | 0.54 |

**On test data:**

- **Random forest**

| Dataset | Specificity | Precision | Recall | Balanced.Accuracy | AUC | MCC |
| --- | --- | --- | --- | --- | --- | --- |
| RWR down | 0.97 | 0.41 | 0.11 | 0.54 | 0.54 | 0.15 |
| **RWR up** | **0.97** | **0.45** | **0.12** | **0.55** | **0.55** | **0.17** |
| RWR non | 0.97 | 0.41 | 0.12 | 0.54 | 0.54 | 0.16 |
| SHP down | 0.96 | 0.27 | 0.07 | 0.52 | 0.52 | 0.07 |
| SHP up | 0.97 | 0.45 | 0.11 | 0.54 | 0.54 | 0.15 |
| SHP non | 0.97 | 0.36 | 0.09 | 0.53 | 0.53 | 0.1 |
| Topo | 0.97 | 0.32 | 0.1 | 0.53 | 0.53 | 0.11 |
| Clique | 0.96 | 0.23 | 0.05 | 0.5 | 0.5 | 0.01 |
| Cluster | 0.97 | 0.41 | 0.08 | 0.53 | 0.53 | 0.1 |
| Sign prot | 0.97 | 0.36 | 0.08 | 0.52 | 0.52 | 0.09 |
| Sign Gene | 0.97 | 0.36 | 0.08 | 0.52 | 0.52 | 0.09 |
| All feature | 0.97 | 0.36 | 0.11 | 0.54 | 0.54 | 0.14 |

- **SVM**

| Dataset | Specificity | Precision | Recall | Balanced.Accuracy | AUC | MCC |
| --- | --- | --- | --- | --- | --- | --- |
| RWR down | 0.95 | 0.26 | 0.36 | 0.66 | 0.66 | 0.27 |
| **RWR up** | **0.95** | **0.3** | **0.45** | **0.7** | **0.7** | **0.34** |
| RWR non | 0.95 | 0.23 | 0.32 | 0.64 | 0.64 | 0.23 |
| SHP down | 0.97 | 0.12 | 0.09 | 0.53 | 0.53 | 0.07 |
| SHP up | 0.95 | 0.11 | 0.14 | 0.54 | 0.54 | 0.08 |
| SHP non | 0.95 | 0.07 | 0.09 | 0.52 | 0.52 | 0.03 |
| Topo | 0.98 | 0.29 | 0.23 | 0.6 | 0.6 | 0.23 |
| Clique | 0.92 | 0.07 | 0.14 | 0.53 | 0.53 | 0.04 |
| Cluster | 0.97 | 0.16 | 0.14 | 0.55 | 0.55 | 0.11 |
| Sign prot | 0.99 | 0.12 | 0.05 | 0.52 | 0.52 | 0.05 |
| Sign Gene | 0.9 | 0.04 | 0.09 | 0.49 | 0.49 | -0.01 |
| All feature | 0.98 | 0.35 | 0.27 | 0.62 | 0.62 | 0.28 |

- **Naïve Bayes**

| Dataset | Specificity | Precision | Recall | Balanced.Accuracy | AUC | MCC |
| --- | --- | --- | --- | --- | --- | --- |
| **RWR down** | **0.92** | **0.14** | **0.27** | **0.6** | **0.6** | **0.14** |
| RWR up | 0.79 | 0.09 | 0.45 | 0.62 | 0.62 | 0.12 |
| RWR non | 0.79 | 0.09 | 0.45 | 0.62 | 0.62 | 0.12 |
| **SHP down** | **0.69** | **0.09** | **0.64** | **0.66** | **0.66** | **0.14** |
| SHP up | 0.71 | 0.08 | 0.55 | 0.63 | 0.63 | 0.12 |
| SHP non | 0.73 | 0.08 | 0.5 | 0.62 | 0.62 | 0.11 |
| Topo | 0.86 | 0.1 | 0.36 | 0.61 | 0.61 | 0.13 |
| Clique | 0.99 | 0 | 0 | 0.49 | 0.49 | -0.02 |
| Cluster | 0.92 | 0.05 | 0.09 | 0.51 | 0.51 | 0.01 |
| Sign prot | 0.98 | 0 | 0 | 0.49 | 0.49 | -0.03 |
| Sign Gene | 0.91 | 0.12 | 0.27 | 0.59 | 0.59 | 0.13 |
| All feature | 0.79 | 0.09 | 0.45 | 0.62 | 0.62 | 0.12 |

- **K-nearest neighbors**

| Dataset | Specificity | Precision | Recall | Balanced.Accuracy | AUC | MCC |
| --- | --- | --- | --- | --- | --- | --- |
| RWR down | 0.97 | 0.21 | 0.18 | 0.58 | 0.58 | 0.16 |
| RWR up | 0.97 | 0.32 | 0.27 | 0.62 | 0.62 | 0.26 |
| **RWR non** | **0.98** | **0.4** | **0.27** | **0.63** | **0.63** | **0.31** |
| SHP down | 0.98 | 0.25 | 0.18 | 0.58 | 0.58 | 0.18 |
| SHP up | 0.99 | 0.14 | 0.05 | 0.52 | 0.52 | 0.06 |
| SHP non | 0.99 | 0.36 | 0.18 | 0.58 | 0.58 | 0.23 |
| Topo | 0.98 | 0.38 | 0.23 | 0.61 | 0.61 | 0.27 |
| Clique | 0.99 | 0 | 0 | 0.5 | 0.5 | -0.02 |
| Cluster | 0.99 | 0.25 | 0.09 | 0.54 | 0.54 | 0.13 |
| Sign prot | 0.97 | 0.13 | 0.09 | 0.53 | 0.53 | 0.08 |
| Sign Gene | 0.98 | 0.2 | 0.14 | 0.56 | 0.56 | 0.13 |
| All feature | 0.98 | 0.33 | 0.23 | 0.6 | 0.6 | 0.25 |

- **Neural Network**

| Dataset | Specificity | Precision | Recall | Balanced Accuracy | AUC | MCC |
| --- | --- | --- | --- | --- | --- | --- |
| **RWR down** | **0.98** | **0.33** | **0.23** | **0.6** | **0.6** | **0.25** |
| RWR up | 0.99 | 0.14 | 0.05 | 0.52 | 0.52 | 0.06 |
| RWR non | 0.99 | 0.4 | 0.09 | 0.54 | 0.54 | 0.17 |
| SHP down | 0.97 | 0.12 | 0.09 | 0.53 | 0.53 | 0.07 |
| SHP up | 0.99 | 0.12 | 0.05 | 0.52 | 0.52 | 0.05 |
| SHP non | 0.97 | 0.07 | 0.05 | 0.51 | 0.51 | 0.02 |
| Topo | 0.88 | 0.14 | 0.41 | 0.65 | 0.65 | 0.18 |
| Clique | 0.26 | 0.04 | 0.73 | 0.49 | 0.49 | -0.01 |
| Cluster | 0.76 | 0.07 | 0.41 | 0.59 | 0.59 | 0.08 |
| Sign prot | 0.59 | 0.07 | 0.64 | 0.61 | 0.61 | 0.1 |
| Sign Gene | 0.84 | 0.04 | 0.14 | 0.49 | 0.49 | -0.01 |
| All feature | 0.99 | 0.29 | 0.09 | 0.54 | 0.54 | 0.14 |
